# Vernalization of Winter Crops Increases Photosynthetic Energy Conversion Efficiency and Seed Yield

**DOI:** 10.3390/plants14152357

**Published:** 2025-07-31

**Authors:** Norman P. A. Hüner, Alexander G. Ivanov, Beth Szyszka-Mroz, Leon A. Bravo, Leonid V. Savitch, Marianna Krol

**Affiliations:** 1 Department of Biology, Western University, 1151 Richmond St., London, ON N6A 3K7, Canada; aivanov@uwo.ca (A.G.I.); bszyszk@uwo.ca (B.S.-M.); mkrol1410@gmail.com (M.K.); 2 Institute of Biophysics and Biomedical Engineering, Bulgarian Academy of Sciences, 1113 Sofia, Bulgaria; 3 Department of Agricultural Sciences and Natural Resources, Universidad de La Frontera, Temuco 4811230, Chile; leon.bravo@ufrontera.cl; 4 Ottawa Research and Development Centre, Ottawa, ON K1A OC6, Canada; leonid.savitch@agr.gc.ca

**Keywords:** vernalization, photostasis, energy conversion, *CBFs*, seed yield

## Abstract

We summarize our present knowledge of the regulation of photostasis and photosynthetic performance versus photoprotection in response to vernalization and conclude that the enhanced photosynthetic performance of winter crops is due to an inherent increase in photosynthetic energy conversion efficiency induced by vernalization which translates into high seed yield in the field as well as under controlled environment conditions. This is consistent with the published data for enhanced photosynthetic performance of the only two extant terrestrial angiosperms, *Colobanthus quitensis* and *Deschampsia antarctica*, native to the frigid conditions of terrestrial Antarctica. The Cold Binding factor family of transcription factors (CBFs/DREBs) governs the enhanced photosynthetic performance of winter cereals as well as the Antarctic angiosperms. In contrast to winter crops, spring varieties survive cold environments by stimulating photoprotection at the expense of photosynthetic performance like that observed for green algae and cyanobacteria. Consequently, this minimizes the photosynthetic energy conversion efficiency of spring varieties and limits their seed yield upon cold acclimation. This review provides critical insights into the regulation of photostasis and the balance between photosynthetic performance and photoprotection in plants and how vernalization has enhanced photosynthetic energy conversion, which is essential for understanding plant adaptation to cold environments and optimizing agricultural productivity for improving crop resilience and yield in challenging climates.

## 1. Introduction

Presently, published data indicate that world crop productivity must increase by at least 50% by 2050 to feed the ever-increasing global human population [1,2,3,4,5,6,7,8]. A major challenge to attaining this goal is the simultaneous environmental stresses projected to occur during unprecedented global climate change conditions in the next 50 to 100 years [9]. This will limit the rate of any further increase in seed yield generated in cereal crops introduced during the green revolution of the 1960s [1,2,3,4,5,9]. However, in addition to providing food for humans and feed for animals, agriculture also contributes significantly to anthropogenic global warming [9]. It is estimated that 25% of global anthropogenic greenhouse gas emissions are due to agricultural production, in part, because of the continued destruction of diverse ecosystems, which act as essential, natural CO_2_ sinks [9,10]. This trend reflects our attempts to maximize crop production and food security by increasing available land for agricultural production [2,8,11].

## 2. Photosynthetic Energy Conversion Efficiency vs. Photoprotection

The link between the process of photosynthesis, biomass production, and seed yield is an extremely complex phenomenon [1,4,6,9,11,12]. A major challenge in our attempts to improve photosynthesis for crop production will be to balance the efficiency of light energy absorbed with the capacity to utilize this energy to convert the fixed CO_2_ into plant biomass energy (photosynthetic energy conversion efficiency defined as (grams leaf biomass/μmol photons s^−1^), PECE) and subsequently convert this biomass energy into increased seed yield [4,7,13]. Photosynthesis couples very rapid, temperature insensitive, photophysical and photochemical processes with the much slower and temperature sensitive biochemical redox reactions involved in electron transport, CO_2_ assimilation, respiration, photorespiration, as well as carbohydrate and nitrogen metabolism. Consequently, plants are typically exposed to excessive excitation energy (EEE) which causes an imbalance between light energy absorbed by the photosynthetic apparatus versus energy utilized by metabolic sinks that govern growth, development, and reproduction [14,15]. Such an imbalance can lead to photoinhibition of photosynthesis, damage to the photosynthetic apparatus, and decrease crop yield [16,17,18,19,20,21,22,23]. To survive such stressful conditions, photosynthetic organisms have evolved photoprotective mechanisms to *dissipate* absorbed light energy in excess of that required for photosynthesis either through non-photochemical antenna quenching (NPQ) [24,25,26], reaction centre quenching [27,28,29,30], and/or the use of alternative electron consumption processes, such as either photorespiration [31], chlororespiration, or mitochondrial respiration, via either the plastid terminal oxidase (PTOX) or the alternative oxidase (AOX) of mitochondria, respectively [32,33]. Collectively, these processes provide photoprotection, safeguarding the photosynthetic apparatus from photodamage and minimizing photoinhibition [1,4,9,11,19,33,34,35].

Although photoprotection from EEE enhances plant fitness [36], to maximize crop productivity under environmental stress conditions, it is assumed that a decreased reliance on NPQ-dependent photoprotection from EEE is required in favour of an increased efficiency and capacity in the utilization of the photosynthetically generated electrons for carbon reduction and biomass production. This could be accomplished in cold-acclimated and winter plant varieties by replacing NPQ-dependent photoprotection with more efficient quenching processes, such as PSII- [28,29] and PSI-dependent reaction center quenching of EEE [37], upregulation of PSI-driven cyclic electron flow (CEF) [33,37], and/or enhanced capacity for CO_2_ assimilation by the chloroplast Calvin–Benson–Bassham (CBB) cycle through increases in the amounts as well as through higher catalytic activities of key regulatory enzymes of carbon metabolism [15,38]. This can be achieved by several non-exclusive mechanisms, such as metabolic regulation by allosteric regulation [39], by the expression of more robust isoforms, or by the induction of a more efficient enzyme turnover [40]. This increases the flux of carbon to metabolic end products, such as sucrose and fructans, in the leaves of winter cereals followed by their subsequent export to the crowns [41,42,43,44,45]. The binding of the CBB protein, CP12, to the chloroplast redox regulator, NADPH dependent thioredoxin reductase (NTRC), is governed by the chloroplast redox state as well as low temperatures [38]. This cold acclimated increase in the capacity of the CBB cycle is associated with the plant vernalization response and is reflected in increases in specific biomass per unit leaf area (SLA), as well as enhanced water use efficiency in leaves of cold acclimated winter cereals [46,47,48]. Furthermore, the potential negative impacts of feedback limited photosynthesis due to an imbalance between source and sink activities [49,50,51] is mitigated in vernalized winter varieties but not spring cultivars [41,42,43]. This enhanced photosynthetic performance of cold acclimated winter cultivars is maintained even under the elevated ambient CO_2_ levels predicted to occur because of climate change [52,53].

## 3. Vernalization and Photosynthetic Performance

Vernalization is a developmental process in overwintering plant species that governs the transition from the vegetative phase during growth at low temperatures in the autumn to the flowering and reproductive phase and the seed set during the warmer temperatures in the spring [54,55,56,57,58,59]. Consequently, the vernalization of winter varieties is associated with long-term growth and development at low temperatures. By contrast, spring varieties do not require exposure to low temperatures to flower and to set seed. Photosynthetic cold acclimation in winter crops and other polar plant species that we discuss below is associated with the long-term vernalization process that requires growth and development at low temperatures, which subsequently induces flowering and seed set, rather than short-term exposures to low temperatures, which we consider a stress, and can result in an inhibition of photosynthesis. Normally, winter cereals are seeded in the early fall, develop and grow vegetatively at low temperatures prior to the onset of winter, during which they develop a novel state, called the *cold acclimated state*, needed to survive freezing stress and other extreme winter conditions. The following spring, the overwintering plants flower and set seed governed by the process of vernalization [12,57,58,59,60,61,62,63]. To become maximally cold acclimated, plants must grow and develop over prolonged periods at low temperatures. Simply shifting plants to low temperatures for short periods of time causes an inhibition of photosynthesis and cellular energy flow rather than inducing a stable, cold acclimated state [34,48,60,61,62,63].

Plant cold acclimation induced by vernalization is an emergent property which integrates processes that occur simultaneously at several different levels of complexity: at the molecular, the cellular, the biochemical, the physiological, and finally at the tissue and the whole plant levels [64,65,66,67,68,69,70,71]. Irrespective of this complexity, access to free energy is an absolute requirement. Thus, acclimation and adaptation of the photosynthetic and respiratory processes are essential for plant survival during exposure to environmental stress. However, the mechanisms underlying survival of environmental stress is species and cultivar dependent [34,41,42,43,61,62,63,64,65,66]. For example, winter cultivars, which require vernalization to flower and subsequently to set seed, are much more resistant to cold and freezing stress than spring cultivars of the same species, which do not require vernalization to flower and to set seed [34,61,62,63,64,65,66].

**Figure 1:**
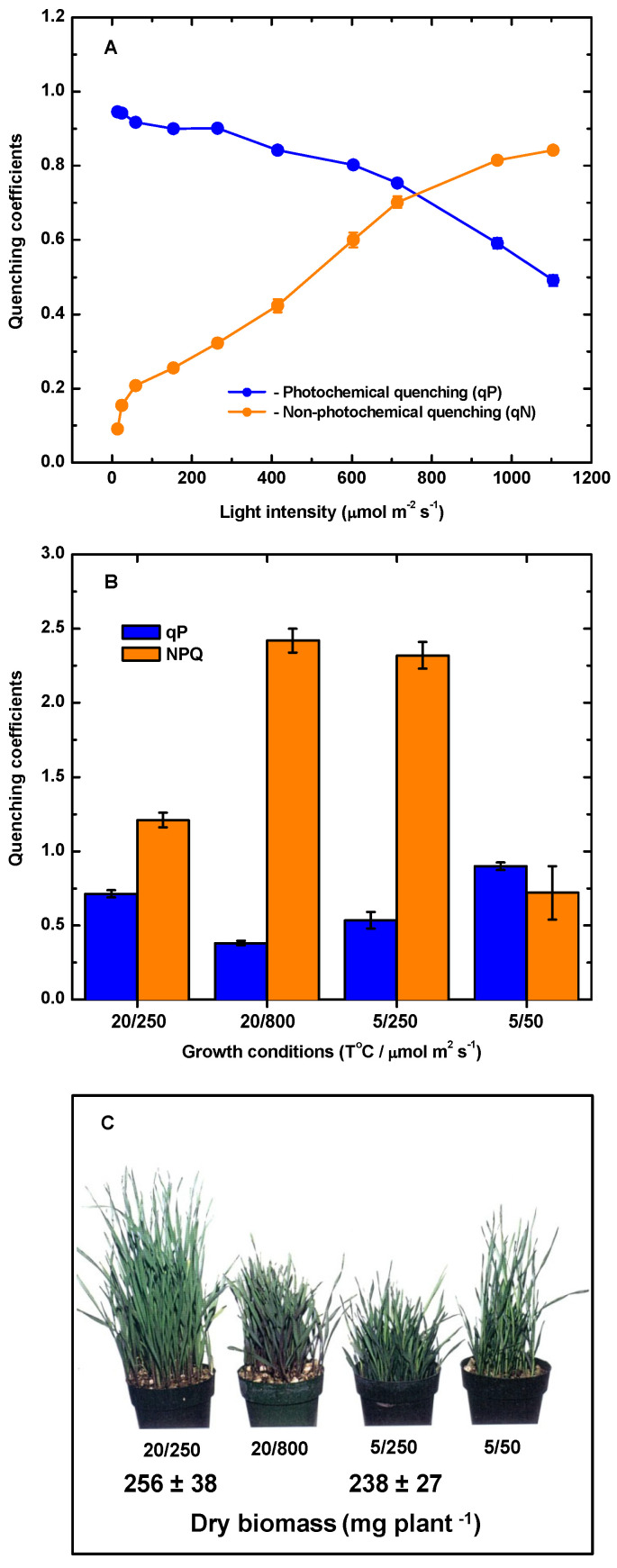
(**A**) Typical photochemical quenching (qP) and non-photochemical quenching (NPQ) light response curves in winter rye *(Secale cereale* L. cv Musketeer) grown at 20 °C and an irradiance of 250 μmol photons m^−2^ s^−1^ and an 8 h/16 h light/dark photoperiod. (**B**) Photochemical quenching (qP) and non-photochemical quenching (NPQ) in *S. cereale* plants were grown at the following conditions 20 °C/250 μmol photons m^−2^ s^−1^, 20 °C/800 μmol photons m^−2^ s^−1^, 5 °C/50 μmol photons m^−2^ s^−1^ and 5 °C/250 μmol photons m^−2^ s^−1^. All presented data are from measurements performed at the corresponding growth temperature and growth irradiance. All values present means *±* SE; n = 3. (**C**) Growth habits and biomass of winter rye plants developed at the temperature/irradiance regimes indicated above. All plants were photographed at similar physiological states based on comparative growth kinetics. All data are taken from [81].

Acclimation to either cold or to high light in winter wheat and rye [12,22,41,42,43,44], spinach [61,62], potato [68], as well as the model plant, *Arabidopsis thaliana* [69,70,71], exhibit an phenotypic plasticity and generate a dwarf phenotype due to tillering (Figure 1) [22,66], which generates an almost two-fold increase in specific leaf area (SLA) and increased leaf absorptance due to elevated chlorophyll accumulation per leaf area compared to spring cultivars [12,60,66]. These phenotypic and morphological characteristics of vernalized, cold acclimated winter cultivars are associated with homeoviscous adaptation [72,73,74,75] with respect to thylakoid membrane lipid and fatty acid composition which affects chloroplast biogenesis, as well as the organization and stability of major photosynthetic Chl-pigment protein complexes involved in the absorption and transformation of light energy in fully expanded leaves [76,77,78,79,80].

A major effect of cold acclimation on winter rye thylakoids is specifically to alter the fatty acid composition of thylakoid phosphatidylglycerol (PG) with respect to its level of the unique plant fatty acid, trans-Δ^3^-hexadecenoic acid [73,74,75]. The lower levels of this unique fatty acid in PG induced by cold acclimation in winter rye is correlated with increased freeze tolerance and the dominance of the monomeric form of LHCII after cold acclimation compared to the dominance of oligomeric form in the non-cold acclimated state [77,78,79]. Furthermore, thylakoid PG in winter rye is specifically bound to LHCII polypeptides rather than a component of the bulk thylakoid membrane lipid pool, and its content of trans-Δ^3^-hexadecenoic acid specifically governs the stability of the supramolecular organization of the major photosynthetic pigment–protein complex, LHCII [73,74,75,76,77,78,79]. These biochemical results for cold acclimation are consistent with changes in chloroplast ultrastructure with respect to smaller thylakoid granal stacks and changes in particle size distributions of PSII–LHCII complexes estimated by freeze–fracture analyses of the thylakoids from cold acclimated versus non-cold acclimated winter rye [80].

We suggest that these factors, along with altered enzyme kinetics, photoprotection, and stomatal conductance all contribute to the stability of the photosynthetic apparatus and enhancement of photosynthetic performance resulting in a 2.0–2.5-fold enhancement of photosynthetic energy conversion efficiency compared to the non-acclimated state (Table 1) because of the re-organization of the photosynthetic apparatus coupled with the increased capacity for the assimilation of CO_2_ in combination with the enhanced export of fixed carbon from source leaves to developmental sinks located in the crown [47,48]. By contrast, spring cultivars normally are less responsive to adjust plant phenotype and photosynthetic energy conversion efficiency in this way in response to growth at low-temperature (Table 1).

**Figure 2:**
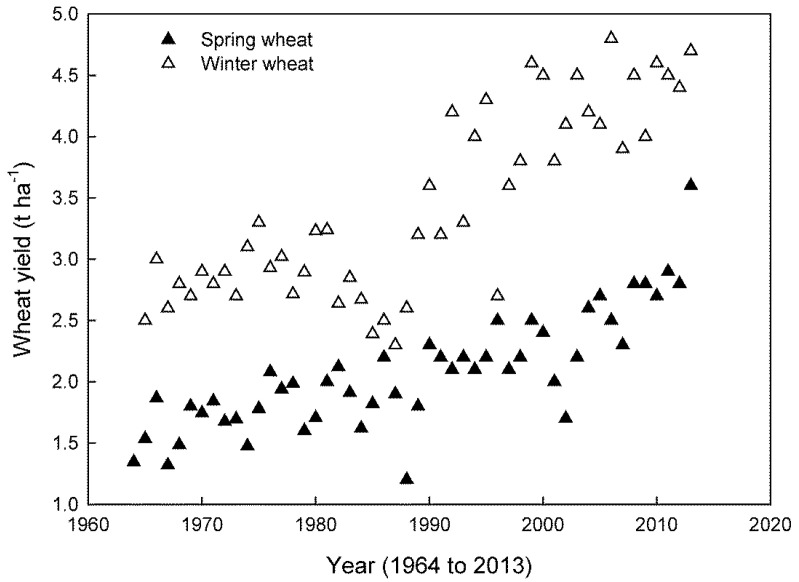
Wheat yields obtained for winter (open diamonds) and spring wheat (closed diamonds) in Canada between 1964 and 2012. Data are from the Statistics Canada website (http://www5.statcan.gc.ca/cansim/pick-choisir (accessed on 12 April 2023)).

Cold acclimation in winter cereals as well as in Antarctic terrestrial plants is governed by the family of transcription factors called C-Repeat Cold Binding factors (CBFs) [82,83,84,85,86,87,88,89,90,91]. In winter wheat, this results in 35–40% higher annual seed yields than in spring wheat under controlled as well as natural field conditions (Figure 2) [12]. The differentially higher seed yields between winter and spring wheat varieties are consistent with data for global winter vs. spring wheat production which favours winter varieties by a considerable margin except for Canada (Table 2). Since spring cultivars do not require vernalization to flower and to set seed, they exhibit reduced photosynthetic performance upon exposure to a low growth temperature and remain very susceptible to low temperature-induced photoinhibition [92,93,94]. Cold acclimation of the Hv*CBF2A* over-expressing line of the spring barley cultivar, Golden Promise, exhibited comparable freezing tolerance to the cold acclimated winter barley, Dicktoo [82]. However, the comparable freezing tolerance in the barley spring cultivar was only detected upon cold acclimation and was not evident constitutively [82]. Clearly, enhancing the freezing tolerance of spring cultivars through overexpression of *CBFs* is possible, which indicates that the process of vernalization is not an absolute requirement to induce freezing tolerance through cold acclimation. However, it remains to be established whether the overexpression of *CBFs* in spring varieties is associated with enhanced photosynthetic performance and seed yield. The results for the Hv*CBF2A* overexpressing line of the spring barley cultivar, Golden Promise, indicate that this may not be the case, since the Hv*CBF2A* over-expressor exhibited reduced total biomass and grain yield compared to the wild-type Golden Promise [82]. Although *CBF* overexpression enhances plant freezing tolerance, the negative effects of *CBF* overexpression on plant development have been noted previously [81,95,96].

Given the complexity of plant development, photosynthesis, regulation of reproduction and seed yield, combined with the projected suboptimal environmental conditions imposed by future climate change, we suggest that it will be an enormous challenge to generate significant and stable increases in crop yield using a molecular approach to redesign the process of photosynthesis, as has been previously suggested [1,4,6,7,9,11]. The observed higher crop yields in winter cereals induced by cold acclimation associated with vernalization is not due to enhanced photosynthetic rates alone, but rather it is due to a co-ordination of molecular events at all levels of complexity, from plant phenotype and plant architecture to leaf morphology, chloroplast ultrastructure [84,87], and reorganization of the photosynthetic apparatus itself [12,97,98]. As has been previously emphasized [4,66], optimizing plant architecture and leaf morphology are critically important to realize the potential positive effects of a molecular redesign of photosynthesis aimed at enhancing crop productivity. It is intriguing to note that, in fact, system-wide changes occur at all levels of complexity in response to vernalization in winter cereals resulting in higher seed yield than in spring wheat, as well as in terrestrial plants adapted to the extreme Antarctic environment [12,34,98,99,100,101,102]. The important general characteristics common to both cold acclimation in winter cereals as well as terrestrial plants native to Antarctica is their inherent flexibility and plasticity reflected in the organization and regulation of their genomes and transcriptomes, which provide the necessary “possibility space” [103] to respond to extreme environmental conditions and generate an exceptional resilience and consequently survive myriad environmental stresses.

Extensive knowledge of photosynthetic acclimation and adaptation to low temperature stress has accrued through the study of a wide variety of non-model species, including diatoms (*Fragilariopsis cylindrus* L.) [98], green algae [104,105,106,107,108,109], brown algae (*Desmarestia menziesii*) [99], vascular angiosperms native to Antarctica, (*Colobanthus quitensis* (Kunth) Bartl. and *Deschampsia antarctica* Desv) [34,100,101,102], woody species native to the Atacama Desert of Chile [110], boreal evergreens [37,111,112], as well as several winter temperate crop varieties of wheat (*Triticum aestivum *L.), rye (*Secale cereale* L.), and canola (*Brassica napus* L.) [12,41,42,43,44,48]. Vernalization-dependent winter cereals consistently exhibit enhanced photosynthetic energy conversion efficiency (Table 1), which is reflected in an increase in specific leaf area (SLA), and is translated into enhanced plant biomass upon the cold acclimation associated with the vernalization process [12,104,108]. An important advantage of this approach is that the vernalization of winter crops as well as plants evolved to sustain the extreme cold Antarctic environment have already optimized their photosynthetic apparatus to ensure their necessary plasticity for long-term survival combined with maximizing their photosynthetic performance in their extreme environments [12,104,113]. This is typically coupled to an array of other adaptive traits, such as the dwarf phenotype, accompanied by increased specific leaf area, increased absorptance and WUE, and enhanced photosynthetic performance. Thus, we suggest that knowledge of photosynthetic acclimation and adaptation associated with vernalization may guide our search for potential avenues through which a combined holistic and molecular approach can not only improve crop productivity but enhance resilience to the environmental stress associated with future climate change.

## 4. Vernalization and Photostasis

Hourly, daily, monthly, and annual fluctuations in photon flux density and light quality, temperature, water, and nutrient availability result in either short-term or long-term disruptions in cellular energy balance or photostasis, and may result in photoinhibition, the light dependent inhibition of photosynthesis due to exposure to excessive excitation energy (EEE) [20,23,114]. Exposure to EEE increases the susceptibility to environmental stress in general [20,22,23,24,26,115,116]. Survival of plants to any environmental stress is dependent upon the plant’s ability to first sense stress-induced energy imbalances and then subsequently mitigate the disruption of photostasis through the activation of the molecular, physiological, and biochemical adjustments needed to re-establish a new, more balanced energy state, called the *acclimated state*, over a broad temporal scale.

Vernalization [54,55,56,57,58,59] not only induces seed set in winter annuals but leads to an indirect re-establishment of photostasis by stable reorganization of the photosynthetic apparatus in response to low temperatures. To maximize plant survival, a dynamic balance must be established between photoprotective energy quenching processes that dissipate EEE to ensure plant fitness versus processes that utilize this energy for growth, maintenance, biomass production, and seed yield [1,4,7,16,22,115,116,117,118,119]. Consequently, from an evolutionary perspective, maximizing plant fitness and survival in a constantly fluctuating environment is of paramount importance rather than maximizing agricultural productivity and seed yield. This is the challenge facing agriculture today and in the foreseeable future.

Fully vernalized winter herbaceous crops, such as wheat, rye, canola, and spinach, as well as native Antarctic species, such as *D. antarctica* and *C. quitensis*, are generally less susceptible to low temperatures and freezing stress than the same species in the non-vernalized state. Furthermore, the capacity to acclimate to any stress is an evolutionary adaptation for survival in a changing environment. Spring varieties, which do not require vernalization to flower, also remain susceptible to cold and freezing temperatures due to their limited plasticity to acclimate to low temperatures compared to winter varieties [12,41,42,43]. Boreal conifers also exhibit differential susceptibilities to temperature stress, in part, due to their inability to increase photosynthetic energy conversion efficiency compared to winter angiosperms [22,37,97,110,111,112,118]. Irrespective of the photoautrophic species, the photosynthetic apparatus plays an essential role in sensing environmental stresses. Thus, it has been proposed that the photosynthetic apparatus has a dual function: not only is it the primary energy transformer for all photoautotrophs, but it is also a primary environmental redox energy sensor [12,120]. Such a dual function is consistent with the “grand design” of photosynthesis first proposed by Daniel Arnon [121] more than 40 years ago.

A retrograde redox signal transmits information regarding the metabolic state of the chloroplasts and mitochondria to the nucleus to induce appropriate changes in the expression of nuclear-encoded photosynthetic, respiratory, and photorespiratory genes to ensure a balanced energy flow to maintain photostasis [122,123,124,125]. The photosynthetic apparatus is essential in governing acclimation and adaptation to environmental stress through cellular redox signalling in cyanobacteria, green algae, and terrestrial plants [126,127,128,129,130,131,132,133,134]. Since sunlight, as a source of energy, enters all photoautrophs through the process of photosynthesis, the photosynthetic apparatus represents a major sensor for the detection of cellular energy imbalances irrespective of the nature of the external stress. This redox sensing mechanism governs the differential expression of complex gene networks to re-establish the cellular energy balance associated with the cold acclimated state. Consequently, sensing an initial stress is a prerequisite to induce a time-nested, photosynthetic acclimation response to a new environment. In addition, photoreceptors also play a prominent role in the regulation of plant developmental responses to changes in temperature [135,136,137,138,139].

During steady-state photosynthesis, changes in photon flux density affect photosynthetic electron transport rates and chloroplast redox energy status which affect the gene expression involved in acclimation to varying light levels, called photoacclimation [122,123,124]. Photoacclimation tailors the structure and composition of the photosynthetic apparatus through retrograde signal transduction pathways to establish cellular energy balance. Pogson et al. referred to the sensing/signalling associated with changes in light quality as “biogenic signals”, whereas the sensing/signalling associated with changes in photon flux density through modulation of the redox state of the PETC are referred to as “operational signals” [140].

## 5. Cold Acclimation, Photostasis and CBFs

The role of photosynthesis in the establishment of maintenance energy through photostasis during plant cold acclimation has largely been ignored historically in studies focussed on plant freeze-tolerance in crop plants and *Arabidopsis thaliana* [88,89,141,142]. However, one unique aspect of plant cold acclimation is that, in some respects, it resembles photoacclimation [12]. This was explained on the basis of the differential temperature sensitivities between the extremely rapid photobiophysical/photochemical and temperature independent processes involved in energy absorption and trapping (energy source) versus the much slower temperature-dependent biochemical and physiological processes that consume this energy (energy sinks) involved in the reduction of C, N, and S in the overall processes of photosynthesis, respiration, photorespiration, and N/S assimilation [12]. This differential temperature sensitivity modulates the redox state of the intersystem PETC like that observed in response to photoacclimation. This low temperature-induced modulation of the chloroplast redox signal not only governs the structure and function of the photosynthetic apparatus, but regulates the phenotypic plasticity observed in cyanobacteria, green algae, and terrestrial plants [12,66,104]. In addition to *CBFs*, the family of dehydration responsive element binding transcription factors (*DREBs*) have been implicated in mediating abiotic stress to dehydration and low temperatures in plants [89,143].

Relative to the non-acclimated state, cold acclimated *Brassica napus* (*Bn*) exhibits higher light-saturated rates of photosynthetic electron transport coupled to higher rates of CO_2_ assimilation like that observed for cold acclimated rye and wheat. This largely accounts for the increased Chl levels and abundances and activities of Rubisco, FBPase, and SPS in winter wheat and winter rye [41,42,43,44,45,46,47,48]. Similar trends for changes in these enzyme activities have been reported for cold acclimation of the Antarctic species, *C. quitensis* [34]. Astonishingly, this low temperature functional response in *Brassica napus* (*Bn*) is mimicked in a *BnCBF3* over-expressor (OE) independent of cold acclimation. Thus, this functional response of the *CBF* over-expressor in *B. napus* is constitutive. In addition, a comparable constitutive response for the expression of the *CBF* homologue (*DaCBF7*) from *D. antarctica* in transgenic rice also resulted in enhanced cold tolerance of the rice [102]. Furthermore, constitutive expression of wheat *CBFs* increased the freezing tolerance of transgenic barley, and overexpression of barley *HvCBF4* not only enhanced the cold tolerance of transgenic rice but decreased its susceptibility to drought and salinity stress [144,145,146,147].

*CBFs* appear to co-ordinate not only system-wide regulation of the dwarf phenotype, freezing tolerance, homeoviscous adaptation, leaf morphology, and plant architecture but the reorganization of the photosynthetic apparatus, enhanced rates of respiration, as well as increased sink capacity in cold acclimated versus non-acclimated plants including winter wheat and winter rye [12,22,66] (Figure 1). These photosynthetic characteristics, as well as growth habit, leaf morphology, and plant architecture are maintained in cold acclimated cereals even when exposed to elevated CO_2_ levels [52,53]. This results in a two-fold increase in photosynthetic energy conversion efficiency in the OE as well as vernalized wheat, rye, and spinach (Table 1). However, this system-wide regulation of photosynthetic performance by *BnCBFs* appears to be gene specific, since overexpression of *BnCBF5* failed to induce this photosynthetic response in *Brassica napus* [148]. Since *CBF*s are present in plants as multigene families [147], a challenge to enhance global food security through vernalization will be to identify the specific member(s) of this multigene *CBF* family associated with the enhanced photosynthetic performance of crop plants.

Shoot-to-root signalling and the regulation of resource allocation is a complex process in plants [149]. Excitation pressure-induced redox signals emanate from the leaf chloroplasts of cold tolerant, terrestrial annuals [12,120], whereas the meristematic regions present in the crown tissue govern tillering and the development of a dwarf phenotype in winter cereals [150,151,152,153]. What is the long-distance signalling pathway linking leaf chloroplast redox status with meristematic crown tissue in cereals that governs tillering and the dwarf phenotype? We proposed a model whereby the low temperature-induced, chloroplast redox signals are initially transmitted to the nucleus through retrograde ROS signalling which induces *CBF* expression [113]. Global transcriptome analyses to test this model in cold acclimated *Arabidopsis thaliana* indicated that, although expression of *AtCBF1–3* are stimulated by low temperature, only *AtCBF3* was specifically enhanced by the redox state of the PQ pool [71]. Furthermore, the global transcriptome analyses indicated that ROS levels appeared to be important in this retrograde signal transduction pathway, which is consistent with previous reports [71]. However, *CBFs* are regulated by myriad environmental stresses, including low temperature, light quality through phytochromes, heat and drought stress, but also by the redox state of the chloroplast PETC [71,154,155,156,157]. If this intracellular, retrograde signal transduction network accounts for the modulation in the expression of nuclear encoded *CBFs* within leaf mesophyll cells, how does the modulation of this *CBF* network affect tillering and the development of a dwarf phenotype?

## 6. Vernalization, Photostasis, and CBFs Converge on Gibberellin Biosynthesis

It is important to appreciate that winter and spring varieties not only exhibit a differential capacity to cold acclimate and tolerate freezing stress [150,151,152,153], but winter wheat consistently exhibits higher seed yields than spring varieties due to its higher photosynthetic energy conversion efficiency (Table 1). From the early 1960s until 2012, annual seed yields of winter wheat varieties exceeded that of spring wheat varieties in the field in Canada by 30–40% (Figure 2) [12]. This was interpreted to reflect the higher potential of cold acclimated winter wheat to enhance photosynthetic capacity and photosynthetic energy conversion efficiency combined with necessary levels of NPQ for photoprotection to maintain photostasis and to enhance survival under extreme growth conditions compared to spring wheat. Thus, photosynthetic acclimation associated with vernalization not only has a significant impact on plant productivity under controlled environment conditions but also under natural field conditions [12].

Gibberellins are a large class of phytohormones that govern the plant phenotype by regulating stem elongation [158,159]. GA3 oxidase is a key enzyme that catalyzes the final step in the biosynthesis of the growth active GA1 and GA4 which stimulate stem elongation [160]. By contrast, mutants that exhibit an impaired GA signalling pathway exhibit a dwarf phenotype due to the accumulation of DELLA proteins which repress the genes involved in stem elongation [161,162,163,164,165,166]. Growth-active GAs bind to DELLA proteins which results in the degradation of DELLA proteins and allows the phytochrome interacting factor, PIF4, to stimulate stem elongation [161,162,163,164]. The net accumulation of growth-active versus growth-inactive GA catabolites is controlled by the relative expression levels and activities of GA2 and GA3 oxidases. Thus, when the activity of GA2 oxidase exceeds that of GA3 oxidase, the level of growth-inactive catabolic GAs exceeds that of growth-active anabolic GAs. Under these conditions, genes required for stem elongation remain repressed due to the presence of DELLA proteins [161,162,163,164,165,166]. Consequently, a dwarf phenotype is generated. The level of endogenous, growth-active GAs appears to be inversely related to *CBF* expression in *Arabidopsis thaliana* [165] and grape leaves [167]. The dwarf phenotype generated by the cold acclimation of winter wheat and rye not only exhibits enhanced photosynthetic performance and water use efficiency but is characterized by at least a doubling in specific leaf area (SLA), which gives rise to an enhanced estimate of photosynthetic energy conversion efficiency (Table 1).

The total shoot biomass of the dwarf, cold acclimated plant can be equal to or greater than that of the non-acclimated, elongated phenotype [41,42,43,44,45,46,47]. This is mimicked by overexpression of *BnCBF17* in *Brassica napus* [48]. Global transcript analyses in *Arabidopsis thaliana* indicate that increased excitation pressure induced by either low temperature or high light enhances GA2ox expression levels and concomitantly modulates GA expression and presumably DELLA protein accumulation [71]. These global transcriptome results are consistent with the inverse relationship between *CBF* expression and growth-active GA accumulation in vernalized *Arabidopsis thaliana* and the induction of a dwarf phenotype and provides insight into the potential mechanism by which the chloroplast redox signals converge on the GA biosynthesis pathway to regulate the dwarf phenotype generated by either photoacclimation or cold acclimation (Figure 1) [71].

The frost tolerance locus, *FR2*, has been mapped to chromosome 5A [168,169,170], and its molecular characterization indicates that this locus includes a complex organization of at least 13 tandemly duplicated *CBFs* [147]. Like the frost tolerance genes located at the *FR2* locus, *VRN1*, the vernalization locus required for the induction of flowering in winter cereals is contiguous with the *FR2* locus and is also activated by low growth temperatures. In winter cereals, *VRN1* is normally repressed and requires exposure to low temperature to be de-repressed and initiate the vegetative to reproductive transition. The activation of *VRN1* appears to negatively regulate the expression of *COR* genes in the *CBF* regulon of the *FR2* locus in cold acclimated winter cereals indicating an interaction between *VRN1* and the *FR2* locus [169]. However, to our knowledge, there is no direct molecular or biochemical evidence yet available to confirm such an interaction between *CBFs* and *VRN1* [151]. The complexity of the regulation of the vernalization process is not restricted to a low temperature-dependence only, since winter cultivars also have a minimal leaf number requirement before entering the reproductive phase. This appears to be governed by the interaction of the photoperiod with the vernalization genes [168,170].

The regulation of winter wheat yields appears to be a consequence of a very complex interaction between the vernalization locus (*VRN1*), the *FR2* locus at which a series of at least 13 *CBFs* are encoded in wheat [147]. Photoperiod genes are important in the control of growth and development after the vegetative to reproductive transition in addition to redox sensitive photosynthetic genes. Presently, there is a major gap in our understanding of the role(s) of *CBFs* in the regulation of the putative molecular interactions between the *VRN1–FR2* loci. Since *CBF* expression is not only linked to increased freezing tolerance, enhanced SLA, the generation of a dwarf phenotype, but also to enhanced photosynthetic performance (Table 1) during vegetative growth in winter cereals, we suggest that future research focussed on the identification of potential *CBF* interactors, as reported by Shi et al. [84] and Kidokoro et al. [171], may provide important insight into the complex mechanism of *FR2–VRN1* interactions and its link to chloroplast redox status, enhanced photosynthetic performance, and seed yield in winter cereals.

Environmental modulation of *CBF/DREB* expression induces broad, pleiotropic effects on plant phenotype, leaf morphology, photosynthetic performance, and stress resilience in *B. napus* [48]. Furthermore, *CBFs* have been reported to respond to various environmental stresses, including heat and drought stress, associated with projected future climate change conditions [8] in various cereals, rice, and vegetable crops. Thus, we suggest that exploitation of the *CBF/DREB* regulons may represent a useful approach to enhancing plant photosynthetic energy conversion efficiency, plant biomass accumulation, plant architecture, and seed yield under the various environmental stress conditions associated with climate change, consistent with the suggestions of Ramirez et al. [34], Byun et al. [102], and Pandey and Senthil-Kumar [172].

**Figure 3:**
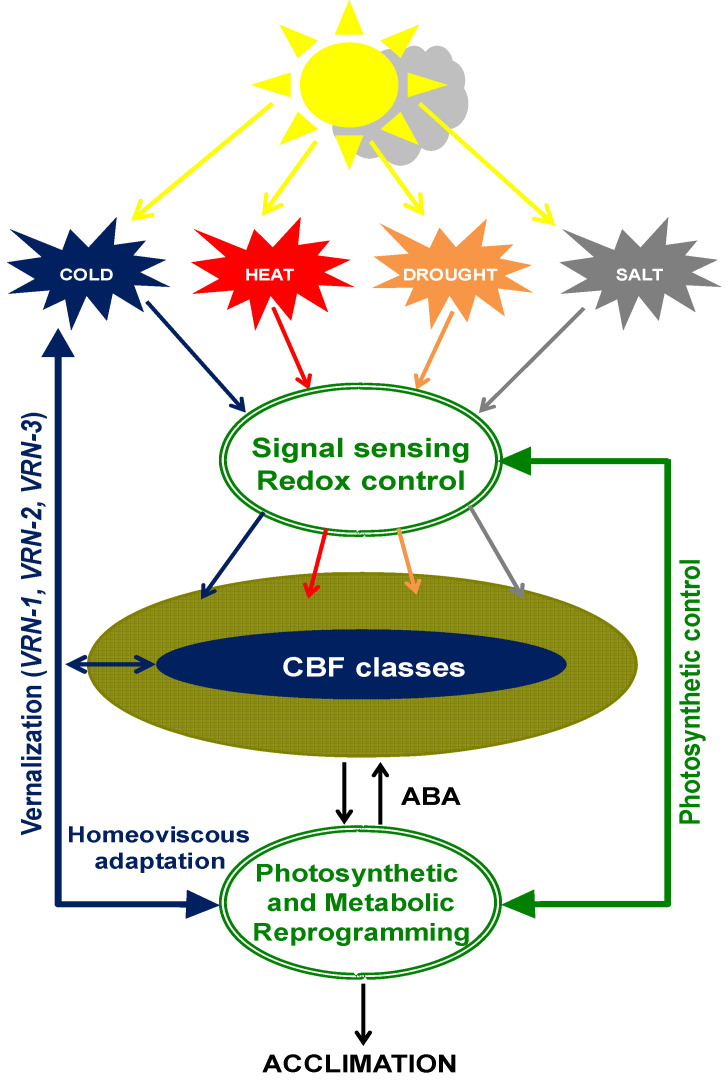
A schematic model of the role of *C-repeat binding factors* (CBFs) for plant responses and acclimation to environmental stresses. The increase in ABA biosynthesis under stress conditions activates *CBF* gene expression which can also be activated independently of ABA, likely directly by chloroplast redox imbalance. Exposure of plants to various stress conditions (cold, heat, drought, salinity) under varying light conditions induces rapid changes in the redox state of the chloroplast photosynthetic electron transport chain and stroma followed by upregulation of specific sets of CBFs responsible for specific photosynthetic and metabolic reprogramming and acclimation to the stress conditions.

Since the establishment of the vernalized state is an emergent phenomenon, we suggest an holistic combined with a molecular approach through the exploitation of vernalized winter crops that have naturally evolved superior fitness, stress resilience, photosynthetic capacity and energy conversion efficiency, and high seed yield combined with myriad other cellular and morphological traits necessary for their survival in a wide array of extreme environments. This combined approach should target the family of C-repeat transcription factors (CBFs) [5,34,85,89,113] to enhance crop productivity, stress resilience, and food security in response to the uncertainty of future climate change conditions (Figure 3). This is supported by the fact that upregulation of *CBF* expression in plants from tropical and subtropical regions, such as millet and king grass, typically grown in the warmer climates also enhanced their stress resilience [173,174]. This has also been accomplished for transgenic rice by the constitutive expression of *DaCBF7* from *D. antarctica* [34,102]. Although this conferred increased tolerance to cold stress in rice, neither its effect on photosynthetic energy conversion efficiency, biomass accumulation, nor its effects on seed yield have been reported. Jeknić et al. [82] reported that, although overexpression of the winter barley Hv-*CBF2A* in spring barley, Golden Promise, enhanced the freezing tolerance of the spring barley, this was associated with a reduction in total shoot biomass and decreased grain yield in the over-expressor compared to the wild type spring barley.

To establish a new dynamic, energy balanced state, redox “operational signals” emanating from the chloroplast represent only one component of a complex intracellular and intercellular sensing/signalling network in plants which integrates redox information in response to changes in light and temperature associated with photosynthesis as well as photoreceptors. Thus, despite the positive effects of vernalization on *CBF* expression and photosynthetic energy conversion efficiency (Table 1), such a positive response appears to be cultivar and species dependent. To overcome the potential negative effects of *CBF* overexpression on plant biomass and grain yield, further research is imperative to minimize the limitation imposed by overexpression of *CBF*s on plant biomass and seed yield.

This review is limited to the effect of vernalization on the direct reorganization of the photosynthetic apparatus at the thylakoid components and how this can affect plant growth and productivity. However, we recognize that vernalization may influence, and vice versa, other components of photosynthesis, such as stomatic responses and carbon assimilation, at the biochemistry level and additionally on improving the antioxidant response of the chloroplasts. These aspects have not been reviewed here, but for a comprehensive review see [175,176,177].

## 7. Conclusions

Projected future climate change conditions associated with myriad environmental stresses, including more frequent and uncertain oscillations in low and high temperatures coupled with decreasing availability of water, will have a significant negative impact on agricultural productivity globally, which will limit our capacity to feed the growing human population [8]. However, the extent of long-term fluctuations in surface air temperatures will continue to vary worldwide from the Polar regions to the north and south temperate regions to the tropical and subtropical regions [8]. Such diverse physical environments with respect to temperature and water availability will expose all plants to increasing environmental stress conditions, which will lower potential crop productivity. However, as discussed above, vernalized winter plant species and crop varieties have developed inherent capacities to mitigate such environmental stresses by maintaining photostasis, photosynthetic energy conversion efficiency, and high biomass accumulation [12]. Thus, a greater focus on vernalized winter crops has the potential to generate higher-than-expected seed yield under stressful conditions such as heat, drought, and low temperature projected to be associated with future climate change. Even with global warming, there still will be regions of the world that will remain amenable to the growth of vernalization-dependent winter cultivars. We suggest that such an holistic approach focussed on winter, vernalization-dependent crops combined with a targeted molecular approach to overexpress *CBFs* in various cereal crops, canola, as well as soybean and vegetable crops, including lettuce and potato, is a relevant avenue for the generation of stress resilient crops combined with potentially increased photosynthetic energy conversion efficiency and increased seed yield to mitigate human food security in response to future global climate change [8]. However, although *CBF* overexpression generally enhances plant cold tolerance and photosynthetic performance, it can have negative effects on seed yield and seed viability, which appears to be cultivar dependent. The molecular basis of such a limitation must be explored to minimize any potential negative effect of *CBF* overexpression. We suggest it would be pertinent initially to survey crop and vegetable cultivars to identify those that exhibit a positive response to *CBF* overexpression with respect to photosynthetic energy conversion efficiency and seed yield.

## 8. Dedication

This review is dedicated to the memory of Andrei Matvejev, Dr. Fergus D.H. Macdowall, and Prof. Gunnar Őquist. Andrei Matvejev, Russian grandfather of NPAH, was an agronomist and plant breeder working in the Soviet Union during the Stalin–Lysenko era. His life’s work was focussed on the enhancement of the cold tolerance and productivity of cereal crops in the former USSR. However, in 1929, he was arrested and imprisoned for 10 years in a Siberian gulag. Despite his imprisonment, he continued his research to improve crop cold tolerance in the Soviet Union. He survived this decade long imprisonment and died in 1975. Dr. Fergus D.H. Macdowall was a research scientist in the Chemistry and Biology Research Institute, Agriculture Canada, Ottawa, Canada, and PhD supervisor of NPAH. He was instrumental in introducing NPAH to the area of plant cold hardiness and supporting his pursuit of the role of photosynthesis in plant cold acclimation. Professor Gunnar Őquist, is a retired faculty member, Department of Plant Physiology, Umeå University, Sweden. NPAH is grateful for his continued friendship and his critically important contributions to ideas and expertise during a life-time of scientific collaborations on photosynthetic acclimation and adaptation to low temperatures.

## Figures and Tables

**Table 1:** Estimated photosynthetic energy conversion efficiency of cold-acclimated and non-acclimated plants (grams leaf biomass/μmol photons s^−1^).

Winter Varieties	Cold Acclimated (CA)	Non-Acclimated (NA)	CA/NA
Wheat cv Norstar	0.288	0.120	2.40
Rye cv Musketeer	0.360	0.148	2.43
Spinach	0.400	0.176	2.27
Brassica WT	0.252	0.108	2.33
Brassica *CBF*-OE	---	0.200	1.85
**Spring Wheat Varieties**			
SR4A	0.160	0.132	1.21
Katepwa	0.168	0.144	1.17

**Table 2:** Total wheat production in 2024 by country (million tonnes), and percentage of winter and spring varieties. Note: data sourced from the Foreign Agricultural Service, USDA (2024).

Country	Total (Mt)	Winter (%)	Spring (%)
China	140	95	5
European Union	121.3	96	4
India	113.3		
Russia	81.5	72	28
United States	53.7	70	30
Canada	35	4	96
Australia	32		
Pakistan	31.4		
Ukraine	22.9		
Turkey	19		

## Data Availability

No datasets were generated or analysed during the current study.

## Abbreviations

ABA, abscisic acid; ABI4, Abscisic acid insensitive transcription factor 4; AOX, mitochondrial alternative oxidase; At, *Arabidopsis thaliana* L; Bn, *Brassica napus* L, C, carbon; CA, cold acclimated; CBF, Cold Binding transcription factor; Chl, chlorophyll; DREB, Dehydration Responsive Element Binding transcription factor; COR, cold responsive gene; EEE, excessive excitation energy; FBPase, fructose bisphosphatase; FR2, frost tolerance 2 gene locus; ΔG, change in free energy; ΔH, change in enthalpy; ΔS, change in entropy; GA, gibberellic acid; LHCII, light harvesting complex II; GUN1, genomes uncoupled 1; I, irradiance; Mg, magnesium; N, nitrogen; NA, non-acclimated; NPQ, non-photochemical quenching; NTRC, NADPH-dependent thioredoxin reductase C; OE, over-expressor; PECE, photosynthetic energy conversion efficiency; PG, phosphatidylglycerol; PGE, photosynthetic gene expression pathway; PETC, photosynthetic electron transport chain; PQ, plastoquinone; PSI, photosystem I; PSII, photosystem II; PTOX, plastid alternative oxidase; SLA, specific leaf area; VRN1, vernalization gene 1; WSC, cell wall integrity and stress responsive component gene.
